# Automated genotyping of microsatellite loci from feces with high throughput sequences

**DOI:** 10.1371/journal.pone.0258906

**Published:** 2021-10-25

**Authors:** Isabel Salado, Alberto Fernández-Gil, Carles Vilà, Jennifer A. Leonard

**Affiliations:** 1 Conservation and Evolutionary Genetics Group, Estación Biológica de Doñana (EBD-CSIC), Seville, Spain; 2 Department of Conservation Biology, Estación Biológica de Doñana (EBD-CSIC), Seville, Spain; University of Goettingen, GERMANY

## Abstract

Ecological and conservation genetic studies often use noninvasive sampling, especially with elusive or endangered species. Because microsatellites are generally short in length, they can be amplified from low quality samples such as feces. Microsatellites are highly polymorphic so few markers are enough for reliable individual identification, kinship determination, or population characterization. However, the genotyping process from feces is expensive and time consuming. Given next-generation sequencing (NGS) and recent software developments, automated microsatellite genotyping from NGS data may now be possible. These software packages infer the genotypes directly from sequence reads, increasing throughput. Here we evaluate the performance of four software packages to genotype microsatellite loci from Iberian wolf (*Canis lupus*) feces using NGS. We initially combined 46 markers in a single multiplex reaction for the first time, of which 19 were included in the final analyses. Megasat was the software that provided genotypes with fewer errors. Coverage over 100X provided little additional information, but a relatively high number of PCR replicates were necessary to obtain a high quality genotype from highly unoptimized, multiplexed reactions (10 replicates for 18 of the 19 loci analyzed here). This could be reduced through optimization. The use of new bioinformatic tools and next-generation sequencing data to genotype these highly informative markers may increase throughput at a reasonable cost and with a smaller amount of laboratory work. Thus, high throughput sequencing approaches could facilitate the use of microsatellites with fecal DNA to address ecological and conservation questions.

## Introduction

Microsatellites are neutral, codominant, highly polymorphic, and abundant genetic markers in eukaryotic genomes [1–3]. Microsatellites are motifs of one to six base pairs repeated in tandem. High polymorphism in these loci yields high information content from only a few markers [4]. These properties have made microsatellites a widely used tool in very different research areas, such as cancer diagnosis [5], human forensics [6], and conservation biology [7]. During the last twenty years, they have also been a powerful and commonly applied tool in molecular ecology for a variety of applications, including individual identification, kinship determination, and population genetics [1–3, 8, 9]. Furthermore, because microsatellite loci are usually short in length, these PCR-based markers can be amplified from low quality DNA samples (e.g. feces or hair) in noninvasive genetic studies [10] as well as for studies involving ancient DNA or museum specimens [11–13].

Microsatellites are generally genotyped through electrophoresis, in which each targeted marker is amplified by polymerase chain reaction (PCR) and then fragment length is determined by capillary or gel electrophoresis [3]. This method is easy to implement, but the number of samples and loci that can be tested in a single run is limited [4]. In addition, this technology is becoming less common in labs, and more difficult to maintain where it remains. An additional problem is that genotypes are difficult to compare across laboratories, or even projects in the same laboratory, which impedes wider data utilization [4].

Next-generation DNA sequencing (NGS) has the potential to overcome some of these drawbacks. The massive parallel sequencing ability considerably enhances throughput, as hundreds to thousands of reads can be generated for many markers and samples simultaneously [14]. NGS may also increase the accuracy of genotyping, because it enables direct access to the nucleotide sequence of amplified microsatellites. Thus, alleles may be identified using the nucleotide sequence and the length of the microsatellite [14]. The potential of NGS to make microsatellite data more comparable across studies and laboratories would greatly enhance their utility. Genotyping microsatellites with NGS would take advantage of their useful traits such as high polymorphism, reduced ascertainment bias when markers are chosen because of the polymorphism at a given population, and high information content [4], while minimizing some of the problems associated with traditional electrophoresis typing such as lack of comparability of results across studies and low throughput.

Manual genotyping of the great number of sequences generated in a single NGS run is seriously time consuming. However, automatic genotyping may now be possible through bioinformatics pipelines. Several software packages have been specifically designed for genotyping amplicons using NGS data: AmpliSAS [15], Megasat [16], MicNeSs [17], and CHIIMP [18]. These packages examine sequences and apply user-definable decision rules to extract potential alleles while discarding amplification or sequencing artifacts (Table 1, for further details on algorithms or software workflow, see original references). Most of the packages have been tested in tissue samples only, although CHIIMP has been evaluated in fecal samples [18]. Noninvasive genetic samples might be a challenge for automatic genotyping software packages as their low DNA quantity and quality increases dropout and produces non-target amplification, increasing genotyping error rates.

**Table 1:** Summary of program properties. Description of programs compared in this study, including an outline of the workflow, default parameters, important features, and brief observations on the ease of use.

Software	AmpliSAS	Megasat	MicNeSs	CHIIMP
**Operating system**	· Web server	· Windows	· Linux	· Windows
		· Mac OS*		· Mac OS*
	· Linux			
				· Linux
		· Linux		
**GUI** †	Yes	Yes	No	No
**Programming language**	Perl	Perl & R	Python 2.7	R
**Workflow**	1. **Demultiplexing** reads into amplicons based on primers sequences.	1. **Demultiplexing** reads into locus-specific files, based on primer and flanking sequences.	1. **Extraction** of the microsatellite with the largest repeat number from all reads of all individuals.	1. **Sample processing** classification of sequences assigning attributes and querying for potential PCR artifacts.
	2. **Clustering** potential alleles and artifacts are grouped together.	2. **Scoring** using sequence depth to obtain genotypes.	2. **Building** observed distribution of the repeat number for each individual.	2. **Genotype calling** filtering and genotyping only sequences that match locus attributes.
	3. **Filtering** separation of artifacts from alleles.	3. **Plotting** histograms of sequence length-frequency distributions for manual verification.	3. **Fitting** by least squares an equal mixture of two discretized asymmetric Gaussians used for all individuals.	3. **Summary and reporting** summary of genotypes and quality control tables and graphs.
**Genotype calling (algorithm)**	All identical reads added to the coverage of the unique variant (‘dominant’ sequence) and variant freq. calculated (if two highly sequenced variants and similar in sequence, ‘subdominant’ seq. considered). In clustering, variants are aligned to each other to find seq. errors, erroneous variants (artefacts) are identified and removed (filtering) and coverages added to the true ones, and a consensus sequence is created (allele asignment). Cluster only exact length/in frame can be user-defined.	Based on depth ratios of as many as four of the most common length variants among amplification products relative to the most common length variant (A1). Decision process considers the relative size (> or < A1) and difference in size of putative alleles relative to A1. If the sum of the two most common sequence length variants exceeds the min. read depth (default = 50), Megasat will score the genotypes. Decision variables are user-definable. Recommended to review genotype calls (depth vs size histogram plots).	Assigning a pair of asymmetric Gaussians from repeat number (that represent alleles, each characterized by four parameters: a mode, substitutions(*s*), a right and left variance) to each individual. Homozygotes have two identical distributions, while for heterozygotes distributions differ, even unequal number of substitutions are considered as different alleles.	Sequences that passes filters (locus’ primer, repeat motif and length range) and exceed min. read depth will be genotyped. Only sequences accounting for at least a min. of the filtered reads are considered (5%). Potential stutters, artifacts or ambiguous sequences are excluded. After filters, if only one sequence remains, then sample labelled as homozygous; if two or more, heterozygous. Several quality control tables and graphs are generated for manually review.
**Default parameters** ‡	· Substitution error rate (%) (clustering) = 1 (Illumina)	· No. mismatches (error tolerance to forward and reverse primers and flanking regions) = 2	· No. substitutions = 1	· Min. read depth = 500
			· Motif size = [2,5]	
	· Indel error rate (%) (clustering) = 0.001 (Illumina)			
			· Min. no. repeats = 4	
		· No. threads (multiprocessing) = 1		
				· Min. no. repeats = 3
	· Min. frequency respect to the dominant seq. (%) (subdominant seq.) (clustering) = 10–25 (Illumina)			
			· Min. read depth = 16	
			· Max. width of the distribution (upper limit for standard deviations) = 5	
	· Min. amplicon depth (no. reads per amplicon) (filtering) = 100			
		**·** Min. read depth = 50		
				· Min. fraction retained of the total no. filtered reads (%) = 5
			· Max. asymmetry of the distribution (ratio between right and left standard deviation) = 2.5	
	· Min. per-amplicon frequency (%) (filtering) = 3			
	· Min. chimera length (filtering) = 10			
	· Max. no. alleles (filtering) = 10 (2, our study)			
**Previous preprocessing steps** §	· Demultiplexing by sample	· Demultiplexing by sample	· Demultiplexing by sample and locus	· Demultiplexing by sample
	· AmpliMERGE		· Format file conversion (fastq -> fasta)	
	· AmpliCLEAN			
				· Adapter trimming (cutadapt)
**Input files (format)** §	· Primer file (TXT, CSV)	· Sequence files (FASTA/FASTQ)	· Sequence files (FASTA)	· Sequence files (FASTA/FASTQ)
				· Sample attributes (CSV)
	· Sequences files (FASTA/FASTQ (R1 & R2 merged))			
		· Primer file & locus attributes (CSV)		
				· Locus attributes (CSV)
				· known individuals (optional) (CSV)
				· Named alleles (optional)(CSV)
**Output files (format)**	· Clustered & filtered sequences (FASTA)·	· Summary of genotypes (TXT)	· Summary of genotypes (CSV)¶	· Summary of genotypes (CSV)·
				Processed files & samples (CSV)
				· Histograms (PNG)
		· Histograms (PDF)		· Allele sequences (FASTA)
	Summary of genotypes (XLS)			
				· Alignments (FASTA)
				· Alignments (PNG)
				· Report (HTML)
**URL**	http://evobiolab.biol.amu.edu.pl/amplisat/	https://github.com/beiko-lab/MEGASAT	https://bioinfo.mnhn.fr/abi/public/micness/	https://github.com/ShawHahnLab/chiimp
**Reference**	15	16	17	18
**Available support for questions/bug reports/suggestions**	Yes, corresponding authors and Google forum available	Yes, corresponding authors	Not possible to contact with corresponding authors	Yes, corresponding authors
**Observations**	· Not intuitive output format.	· GUI† does not work properly in Windows 10·	· It does not work with Python 3	· No need to know R language to run the program, an executable is available after installing required R packages.·
	· AmpliCHECK did not work		· Genotype is not given in the standardize format (length)·	
	· Only possible to change minimum amplicon depth through the command line, not in the web server.			
				Last program version 0.3.1 (31-Jan-2020). Last documentation version (10-Jul-2019)
		Last program version 1.0 (19-Apr-2017). Last documentation version (Dec-2015)	Last program version 1.1 (07-Aug-2015). Last documentation version (11-Aug-2015)	
	· Last program version 1.0 (19-Nov-2018). Last documentation version (24-Jun-2018)			

†GUI: Graphical user interface;

‡ Most important parameters recommended by authors, most of them are user-definable;

§Pre-processing steps and input files used in this study, following the guidelines of authors;

¶ Alleles reported as (mode, substitutions);

*Operating system not tested in this study.

Here we evaluate the ability of several available software packages to genotype microsatellite loci from NGS sequences generated from feces. We compared the performance and ease of use of four software packages for automatic microsatellite genotyping for multiplex-amplified microsatellite loci amplified from fecal DNA of wild Iberian gray wolves (*Canis lupus*). First, we summarize the characteristics of the programs. Second, we evaluated the genotyping success of these packages in terms of genotyping error rates. Third, we assessed the effect depth of coverage has on the genotyping success and estimated the number of reads and replicates needed to reach high confidence in the estimated genotypes.

## Materials and methods

### Materials and lab methods

We collected five apparently fresh Iberian gray wolf (*Canis lupus*) feces in the Cantabrian Mountains of Asturias, northern Spain. The feces samples were collected without any interaction with any animal. We put a small piece of each scat in a 50 ml falcon tube with 70% ethanol. We extracted DNA in an isolated, dedicated laboratory for low quality samples using the QIAmp DNA stool kit (Qiagen) with a negative control. Each extraction and negative was subject to six multiplex PCR reactions (i.e. technical replicates–each reaction was done six times from the same biological sample) including primers to amplify 46 autosomal microsatellites loci previously described in Canids [19–26] (S1 Table in S1 File). This was the first test of these markers as a single multiplex reaction. Reactions contained 1x Phusion Multiplex mix (Thermo Scientific), 0.8 mg/mL bovine serum albumin BSA, 0.05 μM of each primer, and 2 μL of extract in 20 μL and were amplified in a touchdown reaction starting with 98°C for 1 min, then 10 touchdown cycles with 98°C for 10 sec, 67°C to 56°C for 30 sec and 72°C for 30 sec followed by 20 more cycles with the annealing temperature constant at 56°C, ending with a 10 min extension at 72°C and 3 min at 95°C to reduce the formation of primer chains. We checked all products against size standards on agarose gels, and all reactions that yielded product were bead cleaned and dual indexed in a second 12 μL PCR with 1x Kapa HiFi mix (Sigma-Aldrich) and 0.42 μM of each primer (with tail including index and sequencing primer) for 30 sec at 98°C, 12 cycles of 98°C for 10 sec, 60°C for 20 sec and 72°C for 45 sec, and a final 3 min step at 95°C. We checked and quantified products on agarose gels using ImageLab v5.2.1 (BioRad), and pooled equimolar dilutions of all successful PCR reactions for sequencing on a MiSeq (Illumina) with 300 cycles of paired end sequencing at the Johns Hopkins Genetic Resources Core Facility.

### Bioinformatics tools

We independently genotyped microsatellites from the same underlying NGS data with four software packages: AmpliSAS (v1.0) [15], Megasat (v1.0) [16], MicNeSs (v1.0) [17], and CHIIMP (v0.2.2) [18]. CHIIMP was installed and run in Windows 10; MicNeSs, Megasat and AmpliSAS were run in Linux (Ubuntu 18.04.1); and AmpliSAS was also run on the web server (Table 1).

Some pre-processing steps were necessary before performing automatic genotyping (Table 1). Following the AmpliSAT guidelines, we used AmpliMERGE and AmpliCLEAN before AmpliSAS itself to merge paired-end reads and remove reads that did not contain any primer sequences. For MicNeSs, demultiplexing by locus was performed using primer sequences in the “separate by barcode” function in Geneious Prime v2019.1.3 (Biomatters, Auckland, NZ). FASTQ to FASTA format file conversion was also needed for MicNeSs. For CHIIMP, adapters were trimmed using cutadapt [27].

Before the genotyping process, we selected the best microsatellite loci (19 loci), mostly dinucleotides, with alleles ranging in size from 56–172 bp, among all of those initially included for sequencing (46 loci). We discarded microsatellite loci which failed in PCR amplification (i.e. those that did not amplify or only amplified a non-target sequence) in most samples (more than two thirds), loci with very low read depth of the target sequence (less than 16 reads per PCR replicate) in most samples, and compound microsatellites. Compound microsatellites were discarded because not all assessed programs could handle that kind of data—MicNeSs considers only pure repeats (not compound or interrupted).

We used default settings as much as possible to perform genotyping (Table 1). The critical parameter to standardize when comparing programs was the minimum read depth (number of reads) required to call a genotype. Analyses were performed twice for each software: first with the default minimum depth (AmpliSAS = 100 reads; Megasat = 50 reads; MicNeSs = 16 reads; CHIIMP = 500 reads), then with a minimum depth of 16 reads, the lowest default of any of the programs, established by MicNeSs. We used this same, minimum number of reads (16) to make results comparable among software packages. MicNeSs’ genotyping algorithm calculates a Gaussian distribution of the number of repeats that represents each allele, reporting the allele as (*mode*, *s*); being *mode* the mode of the distribution (i.e. largest repeat number) and *s* the number of substitutions in the repeat array [17] (Table 1). To allow comparison with other software packages, we converted genotype results from MicNeSs to allele length (which is the output of the other programs) by considering the number of repeats, the repeat motif and the length of the flanking regions.

### Genotyping error rates

Genotyping of microsatellite loci from fecal DNA is particularly prone to errors [10, 28, 29]. We considered as genotyping error any allelic difference between the genotype obtained from the noninvasive sample using the genotyping software and a reference genotype [30]. Reference genotypes were obtained through consensus of replicates of loci individually scored by the same person (IS) using the lengths graphs function in Geneious Prime (v2019.1.3) on the same NGS data. A second step in the genotyping process was to obtain a reference genotype for all samples and loci. Reference genotypes from manual allele calling of all data were estimated following the criteria: (1) allele must be present at least in 2 PCRs for heterozygotes and 3 PCRs for homozygotes, and (2) homozygotes must not have same second allele in more than 20% of PCRs (Supplementary material, S1 Appendix in S1 File). In just two cases the reference consensus genotype was ambiguous and so were not considered for downstream analyses. We considered allelic dropout (ADO) as the failure to amplify one allele in heterozygous individuals [31, 32] and false allele (FA) as an allele-like artifact that is generated by PCR [33]. Equations (2) and (4) from [31] were used to calculate proportions of ADO and FA, respectively.

We defined the following variables for each locus and each software. *Proportion genotyped* was the number of genotypes estimated by a software pipeline (*Ngenotypes*) divided by the total number of available samples and PCR replicates for the considered locus (excluding the ambiguous genotypes in the reference, see above). *Genotyping success* was the number of genotypes that coincided with the reference (*Nsuccessful*) divided by the total number of genotypes inferred by the software per locus across all replicates and samples. *Proportion ADO* was the number of heterozygote genotypes for which only one of the two alleles could be genotyped (*N*_*ADO*_) divided by the number of heterozygote genotypes in the reference. *Proportion FA* was the number of genotypes including a false allele (*N*_*FA*_) divided by the total number of reference genotypes.

### Statistical analyses

A multidimensional scaling analysis (MDS) was carried out to visualize the similarity between the genotypes generated by the different programs and the reference genotype. A symmetric dissimilarity matrix was obtained from the comparison of consensus genotypes per locus and sample generated by each software when using a minimum read depth of 16 for all programs. We assigned a distance value of 0.5 to ADO and FA (one of the two alleles erroneous), while a distance value of 0 to successful genotypes. Distance matrix included the mean distances values across samples and locus per software (S2 Table in S1 File). Then we performed a two-dimensional ratio MDS, which does not transform input dissimilarities, using the mds function from the package smacof [34] in R version 3.5.2.

We assessed the effect of software and coverage on *Proportion genotyped* and *Genotyping success* when using a minimum read depth of 16 with a generalized linear mixed model (GLMM) with a binomial distribution and using the function glmer from the lme4 R package [35]. Two models were established *a priori* with *Proportion genotyped* and *Genotyping success* as dependent variables. *Software*, *Locus* and *Mean read depth* were included as fixed effects while *Sample* was considered as a random factor. For this analysis, *Proportion genotyped* and *Genotyping success* were calculated for each software, locus and sample, excluding the ambiguous genotypes in the reference (see above). Weights argument were included in each model as the number of trials used to generate each proportion. *Software* and *Locus* were factor variables referring to the genotyping programs and the microsatellite loci. *Mean read depth* was calculated as the mean number of reads of the six PCR replicates per sample and locus (S3 Table in S1 File). *Mean read depth* was centered at the mean and scaled by the standard deviation. The significance of each variable was tested using a Chi-squared test with drop1 function in lme4, which compares the likelihood of the full model including and excluding the variables of interest. When significant differences were found, we also performed pairwise comparisons with Tukey’s post hoc test among programs using emmeans package [36].

We wrote Python, Bash and R scripts (Supplementary material, S2 Appendix in S1 File) to calculate the proportion of correct genotypes for the best performing software when varying the coverage for each PCR product and locus. We used a coverage of 6, 10, 20, 40, 80, 100, 150 and 200 reads. Simulations were run on a dataset with the best quality fecal sample using eleven heterozygous loci with > 200 reads. First, we performed a random subsampling without replacement of reads for each locus and PCR product. Second, those datasets were analyzed using the best performing software. Finally, we evaluated the genotyping success in relation to the number of reads. Simulations were performed with 100 simulation replicates for each locus and PCR product.

We also calculated the number of PCR replicates needed to reach high confidence in a multilocus genotype for the best performing software taking into account the proportion of failed amplifications. As commonly done in noninvasive studies, we considered that a successful genotype must be replicated at least twice if heterozygote and at least three times if homozygote [37]. We first calculated the average proportion of amplifications that resulted in successful genotypes for homozygous and heterozygous loci when using the software with fewer genotyping errors. We then used these proportions to calculate the probability of genotyping success for a single locus after a varying number of replicates using the cumulative probability function of a binomial distribution (probability of obtaining at least 2/3 correct genotypes for a heterozygous/homozygous locus). Finally, we used this value to estimate the probability of correctly genotyping at least 16, 17, 18 and 19 of 19 loci.

## Results

### Sequencing results

A total of 807,084 reads were obtained from the six amplification replicates of the five fecal samples analyzed (mean ± se: 26903 ± 42567 reads per PCR replicate). There was high variance in coverage per locus (from mean ± se: 1 ± 0 (locus 403) to 4154 ± 2165 reads (locus u213), S1a Fig in S1 File). The best 19 autosomal microsatellite loci were selected for downstream analyses (S1 Table and S1b Fig in S1 File).

### Comparison of software performance: Genotyping error rates

**Fig 1:**
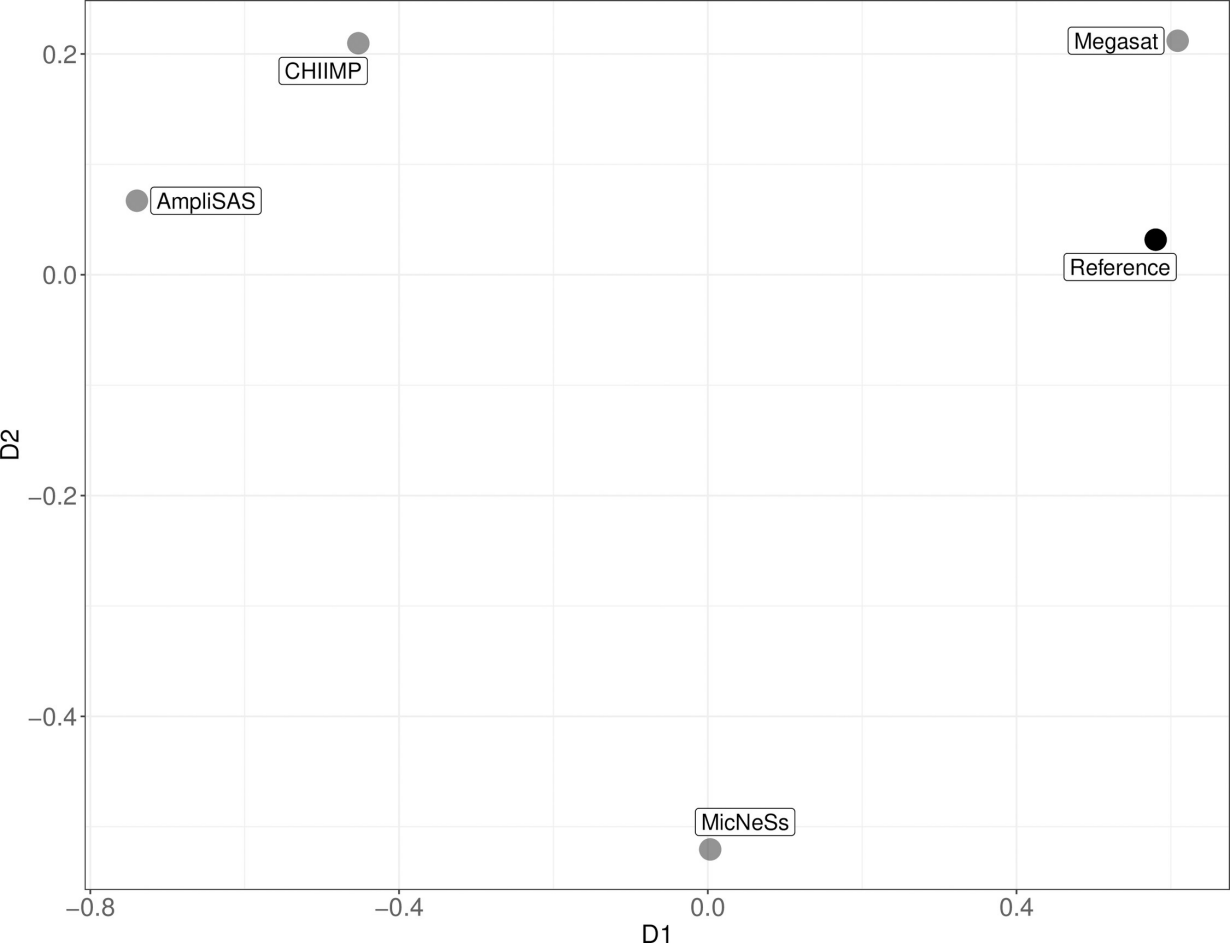
Multidimensional Scaling (MDS) of genotyping programs. Axes show distance values of distance matrix. Distance matrix was obtained from the comparison of consensus genotypes of each software and establishing minimum read depth of 16 (S2 Table in S1 File). The closer two programs are, the more similar their results are. Model statistics: two-dimensions ratio MDS using majorization, stress-1 value (normalized): 0.061, number of iterations: 17. *Reference* indicates consensus genotypes used as reference for comparison.

Software pipelines differed in the proportion of genotypes obtained as output (*Proportion genotyped*) and in the proportion of correct genotypes among them (*Genotyping success*) (Tables 2 and 3). *Proportion genotyped* increased when the minimum read depth was decreased to 16 reads in all programs (except for MicNeSs, for which 16 reads is the default minimum read threshold). However, *Genotyping success* declined with this lower depth threshold. With a standardization of 16 reads, *Proportion genotyped* was generally high for all programs, while *Genotyping success* varied widely (Table 3). Megasat was the software with the highest proportion of correct genotypes, although MicNeSs obtained a greater number of genotypes as output. Megasat had a slightly higher proportion of ADO compared to the other programs. AmpliSAS and CHIIMP showed a very high proportion of FA, with these being present in half or more of the genotypes (Table 3). The comparison of consensus genotypes of each software showed Megasat results were closest to the reference (Fig 1), and that the errors in the genotypes generated from different software occurred from distinct source of bias, as each of them is distant from the others in the plot.

**Table 2:** Genotyping error rates among software packages with default settings. Default minimum read depth; AmpliSAS, 100 reads; Megasat, 50 reads; CHIIMP, 500 reads; MicNeSs, 16 reads. *Proportion genotyped*, the number of genotypes estimated by each software (*N*_*genotypes*_) divided by the total number of reference genotypes per locus across all replicates and samples. *Genotyping success*, the number of genotypes that coincide with the consensus (*N*_*successful*_) divided by the total number of genotypes estimated by the software per locus across all replicates and samples (*N*_*genotypes*_). *Proportion ADO*, the number of heterozygote genotypes for which only one of the two alleles could be genotyped (*N*_*ADO*_) divided by the number of heterozygote genotypes in the reference. *Proportion FA*, the number of genotypes including a false allele (*N*_*FA*_) divided by the total number of reference genotypes. Values are shown as mean ± standard error per locus.

Software	N_genotypes_	N_successful_	N_ADO_	N_FA_	Proportion genotyped	Genotyping success	Proportion ADO	Proportion FA
AmpliSAS	12 ± 1	6 ± 1	0	6 ± 1	0.40 ± 0.04	0.47 ± 0.09	0	0.53 ± 0.09
CHIIMP	5 ± 1	4 ± 1	0	2 ± 1	0.16 ± 0.02	0.69 ± 0.10	0.01 ± 0.01	0.28 ± 0.11
Megasat	15 ± 2	12 ± 2	2 ± 1	2 ± 1	0.50 ± 0.05	0.74 ± 0.08	0.11 ± 0.04	0.15 ± 0.07
MicNeSs	27 ±1	16 ± 2	3 ± 1	8 ± 2	0.91 ± 0.02	0.59 ± 0.06	0.19 ± 0.04	0.31 ± 0.07

**Table 3:** Genotyping error rates among software packages using a minimum read depth of 16. *Proportion genotyped*, the number of genotypes estimated by each software (*N*_*genotypes*_) divided by the total number of reference genotypes per locus across all replicates and samples. *Genotyping success*, the number of genotypes that coincide with the reference (*N*_*successful*_) divided by the total number of genotypes estimated by the software per locus across all replicates and samples (*N*_*genotypes*_). *Proportion ADO*, the number of heterozygote genotypes for which only one of the two alleles could be genotyped (*N*_*ADO*_) divided by the number of heterozygote genotypes in the reference. *Proportion FA*, the number of genotypes including a false allele (*N*_*FA*_) divided by the total number of reference genotypes. Values are shown as mean ± standard error per locus.

Software	Ngenotypes	Nsuccessful	NADO	NFA	Proportion genotyped	Genotyping success	Proportion ADO	Proportion FA
AmpliSAS	25 ± 1	9 ± 2	0	16 ± 2	0.84 ± 0.04	0.36 ± 0.06	0	0.64 ± 0.06
CHIIMP	25 ± 1	11 ± 2	1 ± 0	12 ± 2	0.84 ± 0.03	0.46 ± 0.07	0.07 ± 0.02	0.49 ± 0.06
Megasat	22 ± 1	16 ± 2	3 ± 1	2 ± 1	0.74 ± 0.04	0.72 ± 0.06	0.22 ± 0.05	0.12 ± 0.04
MicNeSs	27 ± 1	16 ± 2	3 ± 1	8 ± 2	0.91 ± 0.02	0.59 ± 0.06	0.19 ± 0.04	0.31 ± 0.07

A GLMM analysis revealed that *Proportion genotyped* was influenced by *Software* (LRT = 72.36, p < 0.001, N = 371), *Locus* (LRT = 139.83, p < 0.001, N = 371) and *Mean read depth* (LRT = 18.82; p < 0.001, N = 371). *Genotyping success* was also influenced by the *Software* (LRT = 172.66, p < 0.001, N = 367) and *Locus* (LRT = 299.08, p < 0.001, N = 367), but not by *Mean read depth* (LRT = 0.58, p = 0.448, N = 367). Post-hoc tests revealed significant pairwise differences among all software packages for *Genotyping success* and for *Proportion genotyped*, except between AmpliSAS and CHIIMP for *Proportion genotyped* (S4 Table in S1 File). Post-hoc tests also showed that, when using the minimum read depth of 16 reads, MicNeSs was the software with the highest *Proportion genotyped*, but Megasat was the software with the highest *Genotyping success* (S4 Table in S1 File). These results suggested that Megasat offered the most consistent results with regard to the reference genotypes generated manually and this program was chosen for subsequent analyses.

### Effect of coverage and number of replicates on genotyping success

**Fig 2:**
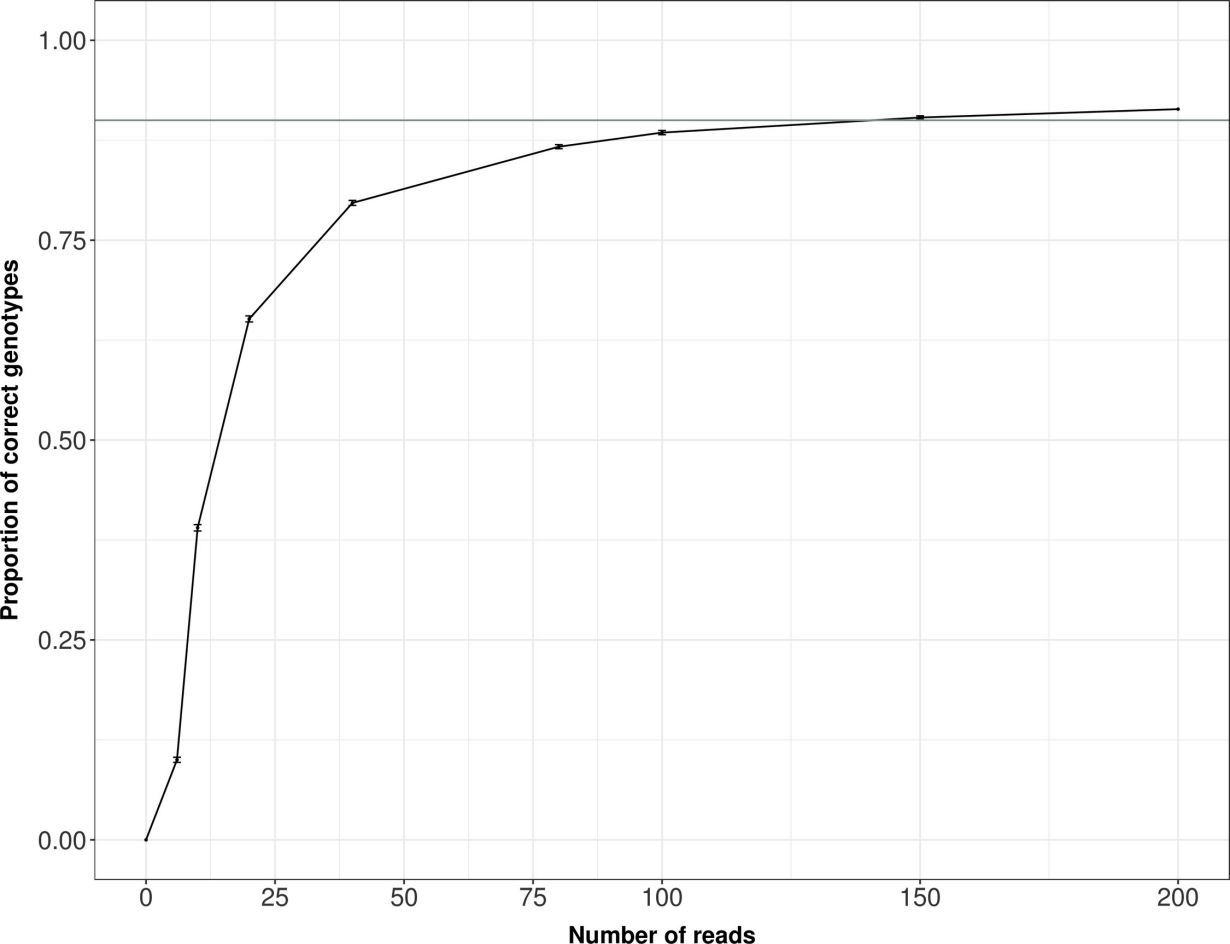
Relation between genotyping success and read depth per locus in Megasat. Proportion of correct genotypes using Megasat when varying the sequencing coverage. With 150 reads *Genotyping success* was greater than 0.9 (horizontal line). Simulations were performed with 100 random draws of a given number of reads for each locus and PCR replicate. The average of 100 random draws is represented; error bars indicate standard errors.

For Megasat, the most reliable software in our analyses, subsampling reads from the genotypes with the largest amounts of data showed that more than about 100 reads per PCR led only to small increases in *Genotyping success* (Fig 2). With 150 reads *Genotyping success* was greater than 0.9 (Fig 2).

**Fig 3:**
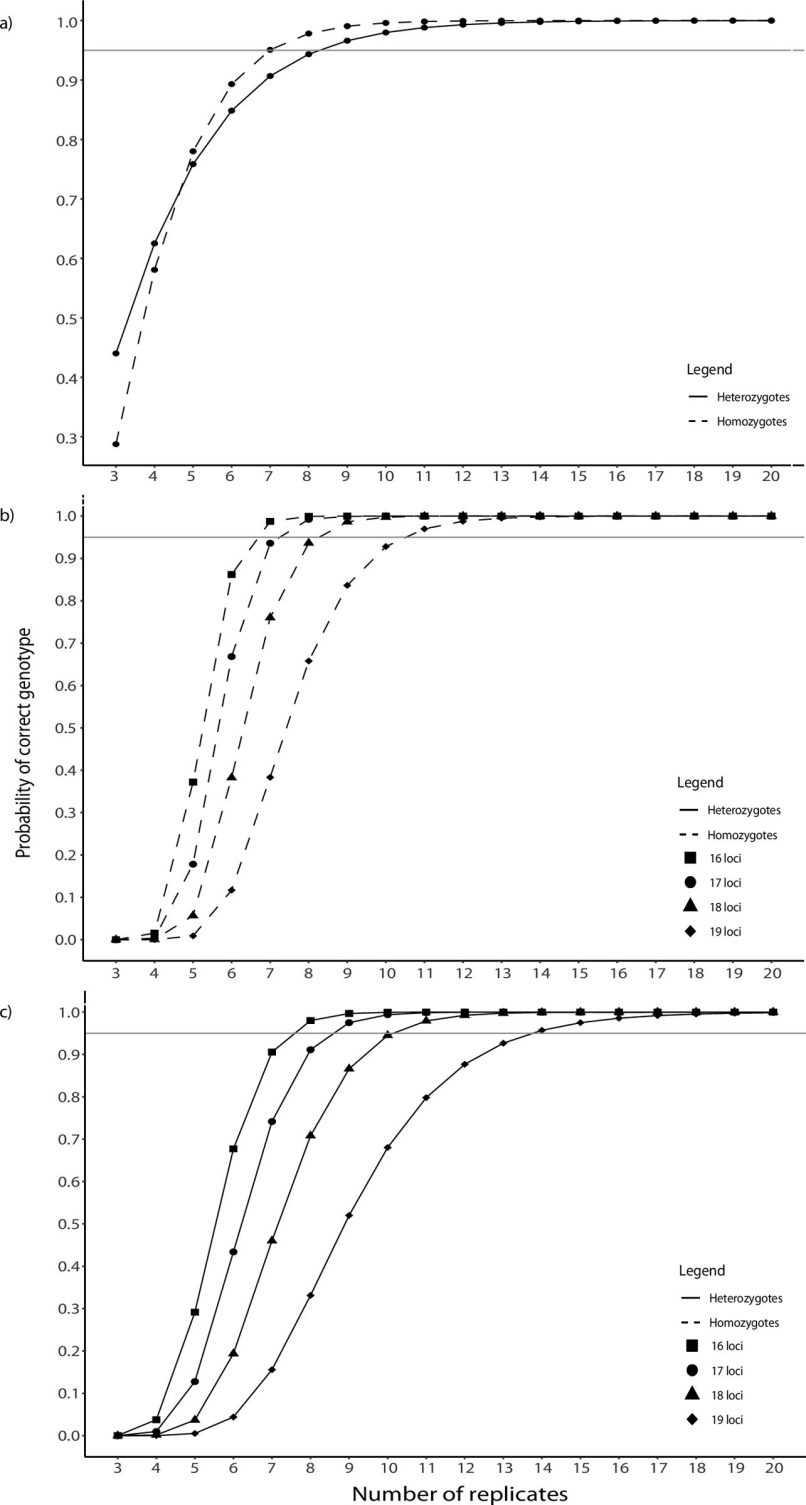
Relation between genotyping success and number of replicates using Megasat. **Single locus (a):** although the probability of genotyping success is higher for homozygotes (0.66; dashed line) than for heterozygotes (0.46; solid line), seven independent replicates are required to determine a homozygous genotype in a noninvasive sample, while eight were for a heterozygote. **Multiple loci (b, c):** Probability of obtaining the correct genotype for multiple homozygous (b, dashed lines) and heterozygous (c, solid lines) loci with different number of replicates. Considering a total of 19 loci, probabilities were calculated to obtain a correct genotype for at least 16 (squares), 17 (circles), 18 (triangles) and 19 (diamonds) of the 19 loci. Horizontal line marks probability of 0.95.

Average rates of genotyping success per PCR in Megasat were 0.46 and 0.66 for heterozygotes and homozygotes, respectively, due to the high ADO rate (Table 3), which can only be detected in heterozygotes. In order to obtain the three positive replicates required to confirm a homozygous genotype or two for heterozygotes, at least seven PCR replicates were needed to assess a homozygote, and eight for a heterozygote, with a probability of 0.95 (Fig 3A). Therefore, it is more difficult to assess a correct heterozygous genotype than a homozygous one.

Taking locus dropout into account, we estimated that to obtain a correct genotype with a probability of 0.95 in 18 of 19 homozygous loci in multiplex, we would need at least eight PCR replicates (Fig 3B). For heterozygotes, we would need at least ten replicates (Fig 3C). This number of replicates is panel-specific, and could be decreased if protocols and marker selection were optimized to reduce locus dropout.

## Discussion

In our evaluation of four different software packages to genotype microsatellites using NGS sequence data, we found differences in performance in terms of proportion of genotypes obtained (*Proportion genotyped*) as well as proportion of correct genotypes (*Genotyping success*). These differences might be due to differences in software design, algorithms and/or filters. While Megasat, MicNeSs and CHIIMP were especially designed for detecting and genotyping microsatellites, AmpliSAS was designed to genotype MHC markers. As MHC markers are generally polygenic in vertebrates [38, 39], AmpliSAS might be biased to call a higher number of heterozygotes, yielding a very low rate of dropout, but a very high rate of false alleles. All packages called substantially higher rates of false alleles than have been reported using traditional microsatellite genotyping of feces via gel electrophoresis [28, 32, 40]. It is not clear if this is an artifact of the sequencing or of the downstream analyses, and should be further explored in future studies.

We attempted to type a large number of markers, but less than half of them were included in the final panel. Locus selection and sample quality play a key role in genotyping success (see also [41]). In this case we employed loci that our group had previously used for other studies on wolves using gel electrophoresis (e.g. [13, 42, 43]). Not surprisingly, many loci that had previously worked with traditional methods failed in this project. It could be because they were not compatible with the multiplex (the multiplex used in this study was very much larger than we ever used for gel electrophoresis due to the limits imposed by few available fluorescent dyes as compared to sequences), or that some loci sequenced better than others. Most of the loci considered here were di-nucleotide repeat microsatellites. It is possible that other, perhaps longer, motifs will sequence better because of reduced strand slippage during PCR [14]. When selecting loci for NGS it is important not only that they amplify in the target organism, and in multiplex if that strategy is used, but also that they are small enough for the largest alleles to be sequenced through. We sequenced on the Illumina MiSeq platform, so our limit was 300 bp.

Sample quality has an important impact on the rate of both allelic dropout and false alleles [44]. In this study all samples were feces, which are generally low-quality samples. However, there is still a lot of variation in quality across fecal samples. This variation could result from the age of the feces when they were collected, weather, or environmental conditions [45–47]. The standard method to overcome the higher rate of genotyping errors associated with the low quality of DNA extracted from feces in the wild has been replications [28, 32]. In some genomic studies higher coverage per locus is used to support the authenticity of a genotype. However, in studies such as this, where it is PCR products and not genomic DNA that is being sequenced, increasing coverage to very high levels should not be considered a replacement for PCR replications. Our results showed that 100 reads per locus maximized information content from the PCR product (Fig 2).

Once a minimum coverage per locus is reached, our results emphasize the importance of including multiple PCR replicates in the analyses, in a similar way to the traditional multi-tube approach [32]. In our case, a minimum of 8 to 10 replicates would be required to ensure reliable genotyping of 18 out of 19 loci (Fig 3). If high reliability at all 19 loci was needed, 11 to 14 replicates could be needed (Fig 3). However, not all research questions have the same requirements, and the number of replicates for a given study could be planned taking into consideration the goals of the study. While robust pedigree reconstructions will require accurate multilocus genotypes, studies about population structure that depend on allele frequencies may need many fewer replicates. The number of replicates required will also depend on the specific panel of loci, target organism, sample type, and level of optimization. Panels can be optimized to lower locus dropout, which would reduce the number of replicates necessary to obtain a genotype. This was the first test of this multiplex reaction, so the large number of replicates necessary for high confidence genotypes could likely be reduced with optimization.

In our analyses, Megasat was the software with the best performance. We tested both the command line and Graphical User Interface (GUI) for Windows versions. We had substantial trouble with the GUI, and chose to do all analyses using the command line. This could be an obstacle to some users, but the command line version is simple, and no advanced programming skills are required to run the program, although a basic knowledge of UNIX commands is. Of the four programs, only AmpliSAS had an operational and online GUI (Table 1), although occasionally the web server had issues refreshing the dataset or some user-defined parameters. Until GUIs are fully implemented in next versions of these programs, we recommend running them through the command line. For technical issues, besides the support provided by the authors, it would be very useful to include a troubleshooting section in the documentations of the programs and a forum for discussion between authors and users in order to promote new versions of the programs with updated documentations and an active support community. AmpliSAS is the only program that has a forum while CHIIMP is the only software which continues releasing new versions with updated documentation.

## Conclusions

Since the advent of high throughput sequencing technologies, a debate has arisen among conservation geneticists about the use of single nucleotide polymorphisms (SNPs) instead of microsatellites. However, the high information content of microsatellites and their applicability in less known systems (where polymorphic loci are not known in advance) make microsatellites useful markers to address a wide range of ecological questions [4]. The primary threat to the long-term utility of these loci is the clearly dated nature of the equipment on which they are generally genotyped, and the lack of comparability between genotypes generated in different studies [48]. The sequencing of these loci on high throughput platforms appears to cure both of these ills. In order to apply this new technology to these markers, protocols for each step of the process from the field to the genotype need to be re-evaluated and made compatible. The number of sequences that come out of NGS sequencers is far beyond what can be visually inspected, as traditional microsatellite genotypes were. In our tests of how to best genotype this data, we find that a relatively low coverage of a reasonable number of replicates can yield automated, high confidence genotypes from fecal DNA. Given the capacity of NGS machines, and automated genotyping, the necessary level of replications should be feasible and should facilitate the higher throughput analysis of feces from wildlife for ecological and conservation studies.

## Supporting information

S1 File (ZIP)
