# Supplementary material for: Automated genotyping of microsatellite loci from feces with high throughput sequences
Source: PLoS One. 2021 Oct 25;16(10):e0258906. doi: 10.1371/journal.pone.0258906 (PMC8544849; doi:10.1371/journal.pone.0258906)
Supplement: S1 File — (ZIP) [file pone.0258906.s001.zip › S1_File.pdf]

Supplementary material. Automated genotyping of microsatellite loci from feces with high throughput sequences

Isabel Salado, Alberto Fernández-Gil, Carles Vilà & Jennifer A. Leonard

TABLES

S1 Table. Autosomal microsatellite loci used in multiplex PCRs. Description of microsatellite loci sequenced in this study, including locus name, location, repeat type, range of allele sizes (asterisk shows size not reported in this study, but in Merllersh et al. (1997) or in the original reference; empty cells (-) show no information available), forward and reverse primer sequences, original reference, and if it was included in the final data set used for downstream analyses. Tails for forward and reverse primers are 5'-TCTTTCCCTACACGACGCTCTT and 5'-GAGTTCAGACGTGTGCTCTTCCG, respectively. (See Methods)

Locus name	Location	Repeat	Allele size (bp)	Forward primer (5'→3')	Reverse primer (5'→3')	Reference	Selected
366	Autosomal	Di	124	ACATCCTCCCTCTAGCACCA	TCCCCACTGCTCATTCTCTT	*Ostrander et al. 1995*	X
377	Autosomal	Di	122-126	ACGTGTTGATGTACATTCCTGC	CCACCCAGTCACACAATCAG	*Ostrander et al. 1995*	X
403	Autosomal	Di	-	TCCTTGTCAGGATTTTGATTCC	AGGAAGGAATATTGACACTTGG	*Ostrander et al. 1995*	
410	Autosomal	Di	56-78	GAGGAAAACCAAGTGATTTTGG	ACCTGCAAGTGACCCTCTCT	*Ostrander et al. 1995*	X
436	Autosomal	Compound	190*	CAGCTGGATTGGGGACTC	CATCTTGCTCTCTCAAC	*Ostrander et al. 1995*	
442	Autosomal	Di	164*	CCAAGAACAGCCTAAGCTGG	ACACATACACGCCCAATTCA	*Ostrander et al. 1995*	
459	Autosomal	Di	-	GGAGCTAGGTAGTGGGACAGG	TGGCATAGTCCCTCTTCCC	*Ostrander et al. 1995*	
466	Autosomal	Di	105-113	TCTGGATTGTGGTCACAACC	ACTGGACACTTCTTTTCAGACG	*Ostrander et al. 1995*	X
474	Autosomal	Di	68-73	TTAAGCCTTATTTTGTGTTGGG	TCCAGGAAGTGTCTGCAGG	*Ostrander et al. 1995*	X
622	Autosomal	Di	209*	CTTGTGCAATCATCATCTTGA	CCCCGAGGTACCTATGGCT	*Ostrander et al. 1995*	
AHT_H171	Autosomal	Di	82-88	CTCACCAGGCATAGACACTCAG	CTCATTTGTTCACGCACCC	*Breen et al. 2001*	X
AHT_H260	Autosomal	Di	245*	CGCTATACCCACACCAGGAC	CCACAGAGGAAGGGATGC	*Breen et al. 2001*	
c2096	Autosomal	Tetra	57-65	CCGTCTAAGAGCCTCCCAG	GACAAGGTTTCCTGGTTCCA	*Francisco et al. 1996*	X
c758	Autosomal	Di	228*	AAGCATCCAGAATCCCTGG	GTTGATTGGGAGATAATCCACA	*Mellersh et al. 1997*	
CPH12	Autosomal	Di	194-216*	GGCATTACTTGGAGGGAGGAA	GATGATTCCTATGCTTCTTTGAG	*Fredholm & Winterø 1995*	
CPH14	Autosomal	Di	182-203*	GAAAGACAATCCCTGAAATGC	ACCCCATTTATGAGAATCATGT	*Fredholm & Winterø 1995*	
CPH16	Autosomal	Di	149-187*	CTACACCAGTTAGGGAATCTAGC	CAGATTCAAATCCACTCTCAGAC	*Fredholm & Winterø 1995*	

CPH17	Autosomal	Di	229-251*	GAGAACAAAAGTCCCATGCAC	GCATTGATGCTAATGCAAATG	*Fredholm & Winterø 1995*	
CPH2	Autosomal	Di	52-58	TTCTGTTGTTATCGGCACCA	TTCTTGAGAACAGTGTCCTTCG	*Fredholm & Winterø 1995*	X
CPH3	Autosomal	Compound	154-182*	CAGGTTCAAATGATGTTTTCAG	TTGACTGAAGGAGATGTGGTAA	*Fredholm & Winterø 1995*	
CPH4	Autosomal	Di	118-150*	ACTGGAGATGAAAACTGAAGATTATA	TTACAGGGGAAAGCCTCATT	*Fredholm & Winterø 1995*	
CPH5	Autosomal	Di	74-88	TCCATAACAAGACCCCAAAC	GGAGGTAGGGGTCAAAAGTT	*Fredholm & Winterø 1995*	X
CPH6	Autosomal	Di	82	CATTGGCTGTTTGACTCTAGG	ACTGATGTGGGTGTCTCTGC	*Fredholm & Winterø 1995*	X
CPH7	Autosomal	Di	124-128	ACACAACTTTCCATAATACTTCCCA	ATCAATGCTCTCCTCCCCAG	*Fredholm & Winterø 1995*	X
CPH8	Autosomal	Di	186-204*	AGGCTCACAATCCCTCTCATA	TAGATTTGATACCTCCCTGAGTCC	*Fredholm & Winterø 1995*	
CPH9	Autosomal	Di	139-155*	CAGAGACTGCCACTTTAAACACAC	AAAGTTCTCAAATACCATTGTGTTACA	*Fredholm & Winterø 1995*	
FH2010	Autosomal	Tetra	228*	AAATGGAACAGTTGCGCATGC	CCCCTTACAGCTTCATTTTCC	*Francisco et al. 1996*	
FH2054	Autosomal	Tetra	151*	GCCTTATTCATTGCAGTTAGGG	ATGCTGAGTTTTGAACTTTCCC	*Francisco et al. 1996*	
FH2097	Autosomal	Tetra	288*	CAATGTCGAATTCCATGGTG	ATGGAGCAAGATGTGTTTGTG	*Francisco et al. 1996*	
FH2109	Autosomal	Tetra	176*	CAATCCAGCAACCCTCATCT	CAGGGATTGAGTCCCACATC	*Francisco et al. 1996*	
FH2130	Autosomal	Tetra	300*	GCTGTCCTGCACTTTTCCTC	GTTAAGGAATAGTTGGGGGTCC	*Francisco et al. 1996*	
Ren37H09	Autosomal	Di	214*	ATTCCCTTGTATTGCTCA	CCCCAAAAAATCCAACCA	*Jouquand et al. 2000*	
Ren49F22	Autosomal	Di	157*	GGGGCTCTGTTATTAGGTG	TCATAAGGCAAAGAAAACC	*Jouquand et al. 2000*	
u109	Autosomal	Di	100-110	AACTTTAAGCCACACTTCTGCA	ACTTGCCTCTGGCTTTTAAGC	*Ostrander et al. 1993*	X
u123	Autosomal	Di	135*	AACTGGCCAAACATAAACACG	TTCATTAACCCTTTGCCCTG	*Ostrander et al. 1993*	
u140	Autosomal	Di	89-101	CAGAGGTGGCATAGGGTGAT	TCGAAGCCCAGAGAATGACT	*Ostrander et al. 1993*	X
u172	Autosomal	Di	114	CCTGTCTCCTGTGGACCAAT	ACATGCAAAAGGACACATTACG	*Ostrander et al. 1993*	X
u173	Autosomal	Di	65-73	ATCCAGGTCTGGAATACCCC	TCCTTTGAATTAGCACTTGGC	*Ostrander et al. 1993*	X
u20	Autosomal	Di	-	AGCAACCCCTCCCATTTACT	TTGATCTGAATAGTCCTCTGCG	*Ostrander et al. 1993*	
u204	Autosomal	Di	164-172	CGAGAGCAACATAGGCATGA	CAAAGTGCTGTGGCAGGTC	*Ostrander et al. 1993*	X
u213	Autosomal	Compound	-	AATATGGGAGAGGAGAAGAGGG	ATGCTTCCTGGTAAGCAATCA	*Ostrander et al. 1993*	
u225	Autosomal	Di	120-124	AGCGACTATTATATGCCAGCG	CTCATTGGTGTAAAGTGGCG	*Ostrander et al. 1993*	X
u250	Autosomal	Compound	91-93	TTAGTTAACCCAGCTCCCCCA	TCACCCTGTTAGCTGCTCAA	*Ostrander et al. 1993*	
u253	Autosomal	Compound	64-68	AATGGCAGGATTTTCTTTTGC	ATCTTTGGACGAATGGATAAGG	*Ostrander et al. 1993*	
u279	Autosomal	Di	83-89	TGCTCAATGAAATAAGCCAGG	GGCGACCTTCATTCTCTGAC	*Ostrander et al. 1993*	X
VWF	Autosomal	Hexa	105	CTCCCCTTCTCTACCTCCACCTCTAA	CAGAGGTCAGCAAGGGTACTATTGTG	*Shibuya et al. 1994*	X

S2 Table. Symmetric dissimilarity matrix used for MDS (Fig. 1). Distance matrix include mean distances values across samples and locus per software.

	Reference	Amplisas	Micness	Megasat	CHIIMP
**Reference**	0	0.24605263	0.15601852	0.06008772	0.20263158
**Amplisas**	0.24605263	0	0.18289474	0.25087719	0.11096491
**Micness**	0.15601852	0.18289474	0	0.17192982	0.16184211
**Megasat**	0.06008772	0.25087719	0.17192982	0	0.20263158
**CHIIMP**	0.20263158	0.11096491	0.16184211	0.20263158	0

S3 Table. Data table used for GLMM analysis to assess the effect of sequencing coverage and software. *Software* is the program used; *Feces,* the sample used; *Locus,* the locus name; *Proportion_genotype*d, the proportion of genotypes estimated by a software pipeline; *Genotyping_success,* the proportion of genotypes that coincided with the reference*; Mean_depth,* the mean sequencing coverage of the six PCR replicates; *Proportion_genotyped, Genotyping success* and *Mean_depth* were calculated per sample, locus and software*; w_genotypes and w_success* are the weights used in each model as the number of trials used to generate each proportion: *w_genotypes,* the number of PCR replicates (for model with *Proportion_genoyped* as a response variable) and *w_success,* the number of genotypes estimated by a software pipeline (for model with *Genotyping _success as dependent variable)*. *Na v*alues marks the ambiguous genotypes in the reference, not considered for the analysis**.**

(Table S3 in excel file)

S4 Table. Pairwise differences among programs. Results of Tukey's post-hoc tests for *Proportion genotyped* and *Genotyping success* with minimum read depth of 16 reads. Values in bold indicates significant p-values (< 0.05). SE, standard error.

	Estimate	SE	Z-ratio	p-value
Proportion genotyped				
AmpliSAS - CHIIMP	0.05	0.19	0.28	0.992
AmpliSAS - Megasat	0.87	0.18	4.89	**< 0.001**
AmpliSAS - MicNeSs	-0.74	0.21	-3.48	**0.003**
CHIIMP - Megasat	0.82	0.18	4.62	**< 0.001**
CHIIMP - MicNeSs	-0.80	0.21	-3.75	**0.001**
Megasat - MicNeSs	-1.62	0.20	-7.91	**< 0.001**
Genotyping success				
AmpliSAS - CHIIMP	-0.49	0.15	-3.24	**0.007**
AmpliSAS - Megasat	-1.92	0.17	-11.46	**< 0.001**
AmpliSAS - MicNeSs	-1.24	0.15	-8.23	**< 0.001**
CHIIMP - Megasat	-1.43	0.16	-8.77	**< 0.001**
CHIIMP - MicNeSs	-0.76	0.15	-5.14	**< 0.001**
Megasat - MicNeSs	0.68	0.16	4.22	**0.001**

FIGURES

S1 Fig. Distribution of coverage among loci. Mean number of reads per locus and PCR replicate across all samples. Error bars indicate standard error. Total reads from the six replicates of the five fecal samples were 807,084. **(a)** All loci included in the multiplex (46). **(b)** Final loci used in downstream analyses (19). We discarded microsatellite loci which failed in PCR amplification in most samples (403, 436, 622, AHT_H260, CPH9, CPH12, CPH14, CPH16, CPH17, FH2097, FH2130, Ren37H09, u123, u213), compound microsatellites (u250, u253), and loci with very low read depth of the target sequence (< 16 reads per PCR) in most samples (442, 459, c758, CPH3, CPH4, CPH8, FH2010, FH2054, FH2109, Ren49F22, u20). Unknown refers to sequences with no primers sequences detected. Black vertical line marks 100 reads.

(a)

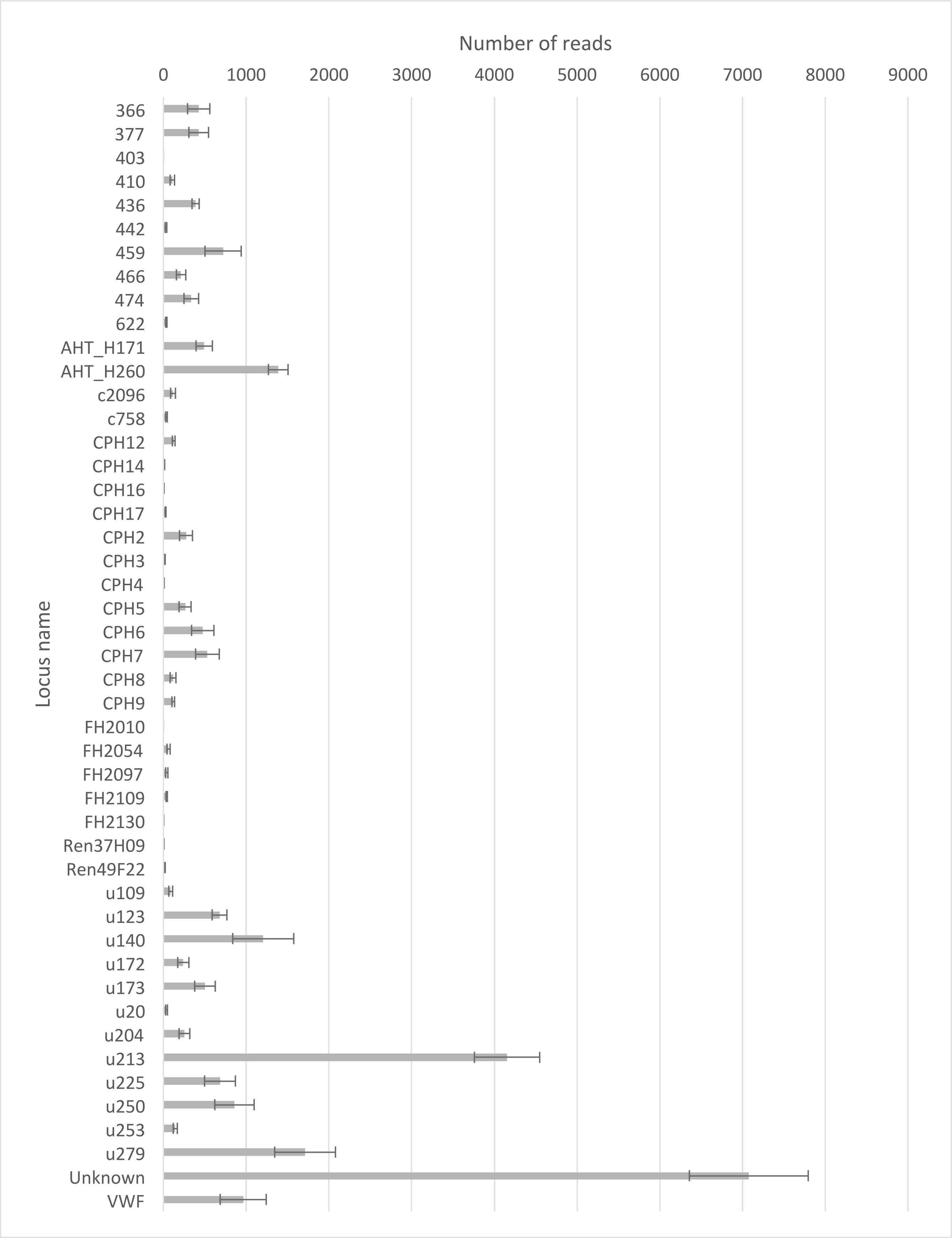
Number of reads
0
1000
2000
3000
4000
5000
6000
7000
8000
9000
Locus name
366
377
403
410
436
442
459
466
474
622
AHT_H171
AHT_H260
c2096
c758
CPH12
CPH14
CPH16
CPH17
CPH2
CPH3
CPH4
CPH5
CPH6
CPH7
CPH8
CPH9
FH2010
FH2054
FH2097
FH2109
FH2130
Ren37H09
Ren49F22
u109
u123
u140
u172
u173
u20
u204
u213
u225
u250
u253
u279
Unknown
VWF

(b)

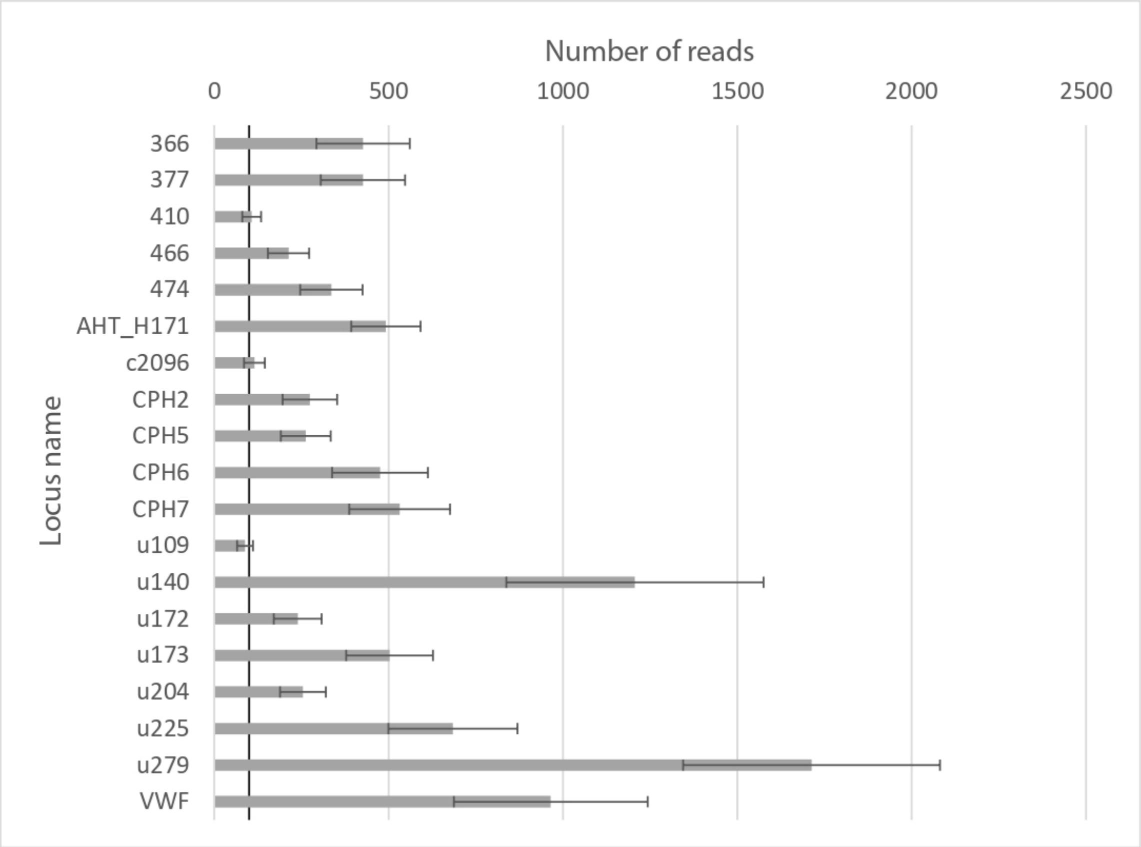

Number of reads
0
500
1000
1500
2000
2500
Locus name
366
377
410
466
474
AHT_H171
c2096
CPH2
CPH5
CPH6
CPH7
u109
u140
u172
u173
u204
u225
u279
VWF

APPENDICES

S1 Appendix - Protocol for manually generating the reference genotype using Geneious Prime 2019

Reference genotypes were obtained through consensus of replicates of loci individually scored by the same person (IS) using the lengths graphs function in Geneious Prime (v2019.1.3) on the same NGS data. A second step in the genotyping process was to obtain a reference genotype for all samples and loci.

1. **Reviewing sequences**

After de-multiplexing by technical replicate (PCR product) and locus, all sequences were visually checked in Geneious.

No genotype was scored (NA) when:

a) If there was not enough read depth per technical replicate and locus (< 16 reads)*, it was considered 'background noise'.
b) If there was no microsatellite repeat in the sequences, it was considered as a 'PCR failure'.
c) If there was an unclear distribution of peaks, it was considered as 'Unscore' (see Appendix S2 (D) in Selkoe & Toonen 2006).

*In this step, several thresholds have been proposed by other authors. Vartia et al. 2016 used individuals with 5 or more reads for a given locus. Brandariz-Fontes et al. 2015 used only cases with more than 10 reads per individual test. We used 16 reads per PCR replicate to standardize the same threshold used to compare between programs.

Examples:

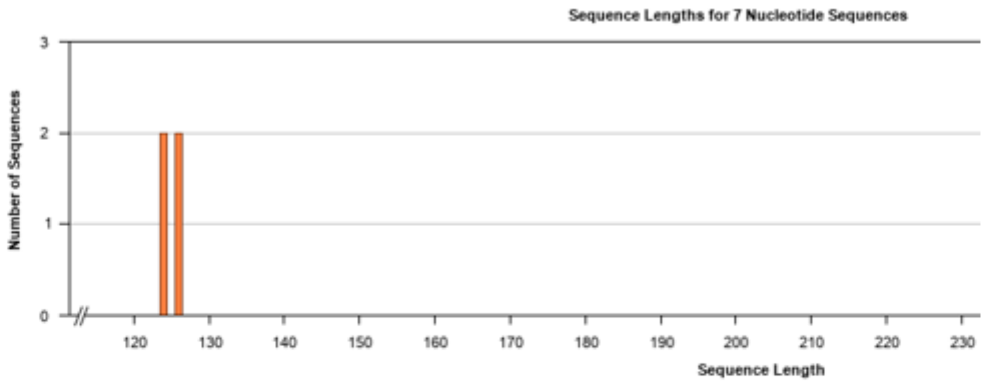

Sample 5866 - PCR 2 - Locus 366: Genotype NA (low read depth)

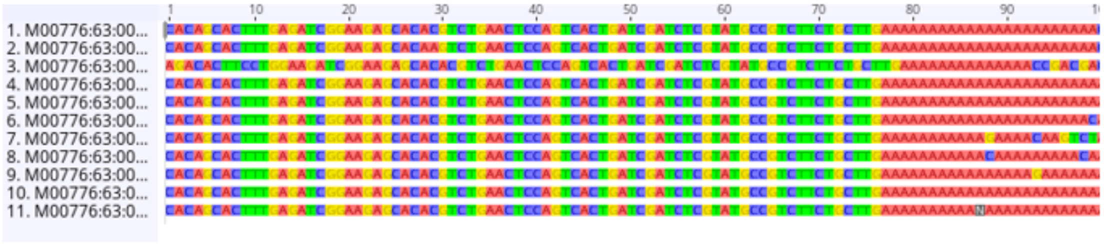

Sample 5866- PCR 4 - Locus 377: Genotype NA (PCR failure)

2. Genotyping technical replicates

Each technical replicate was scored individually by checking the distribution of peaks. Distributions had to be clear for a genotype to be taken into account.

If there were two well-defined big peaks in the distribution, the locus was considered ***heterozygote***. If there was only one big peak, it was considered ***homozygote***.

If two big peaks in the distribution were very close to each other, alleles and stutter patterns were differentiated as:

- If the two peaks only differed in 2 bp (very close to each other), especially common in dinucleotide microsatellites, we looked at the distribution of stutter bands. If the smallest peak (lower number of bp, e.g. 122) is higher (a greater number of reads) than the biggest peak (e.g. 124) and also the pattern of stutter bands is different, then is ***heterozygote*** (similarly as in Appendix S2 (B) in Selkoe & Toonen, 2006).

- On the contrary, if there was a growing distribution of smaller peaks previous to the biggest peak and the pattern of stutter bands is similar, we considered these peaks as stutter or shadow bands, then is ***homozygote.***

Loci 650-79.3 and 990-35 could only be homozygous since they amplify at a region of the Y chromosome. In these cases, only the most frequent sequence length was considered.

Examples:

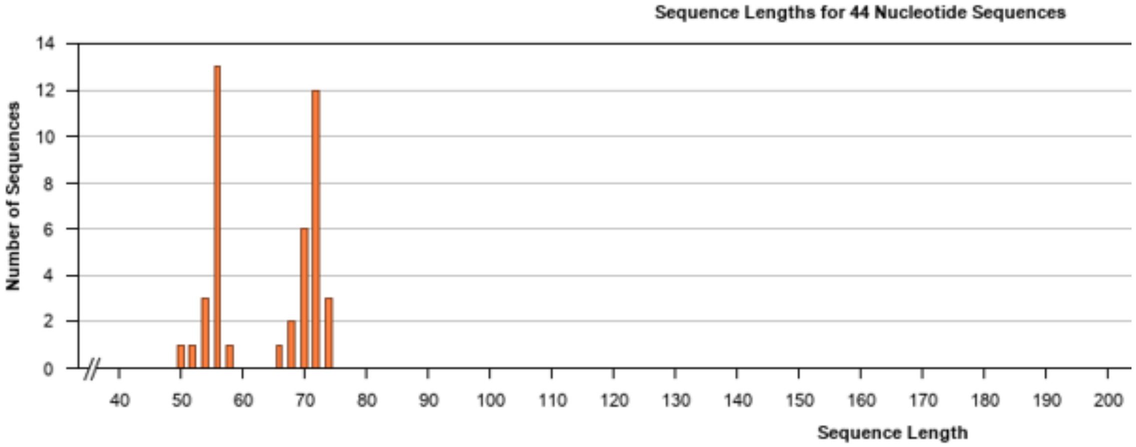

Sample 5870 - PCR 1 - Locus 410: Genotype 56 72

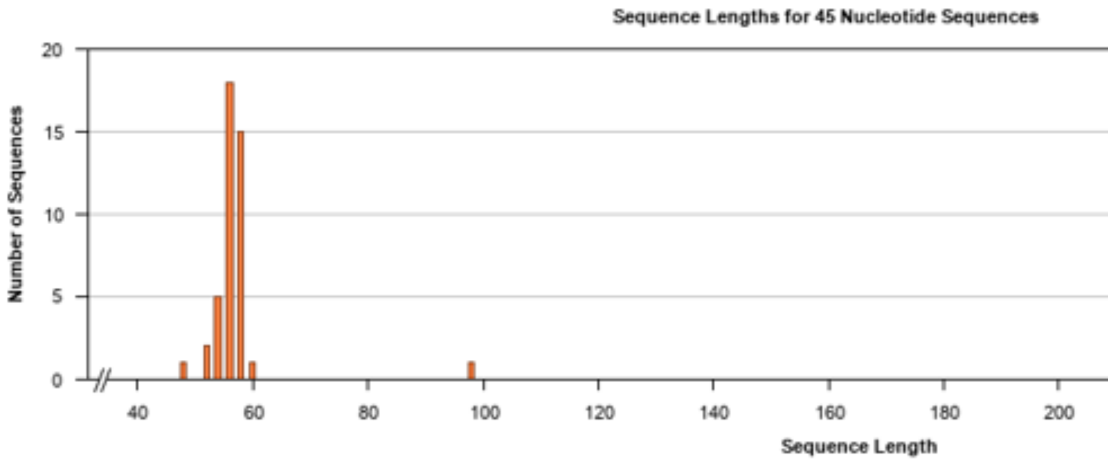

Sample 5866 - PCR 6 - Locus CPH2: Genotype 56 58

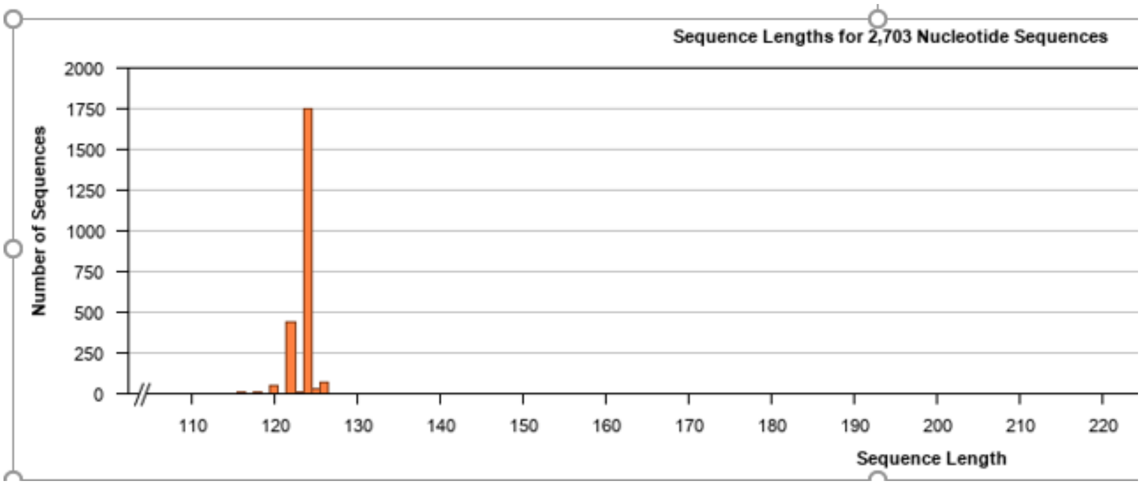

Sample 5867 - PCR 6 - Locus 366: Genotype 124 124

3. **Obtaining the consensus genotype per sample**

To estimate the consensus genotype, genotypes of technical replicates per locus within each sample were compared and distribution of peaks per locus was checked again to identify putative genotyping errors (false alleles or allelic dropout).

Zigosity was specified following the criteria:

1) Allele must be present at least in 2 PCRs for heterozygotes, and 3 PCRs for homozygotes.
2) Homozygotes must not have same other allele in more than 20% of PCRs.

Examples:

Sample-PCR\Locus	366	366-b	377	377-b	CPH2	CPH2-b	CPH6	CPH6-b	u279	u279-b
5866-1	124	124	122	122	58	58	82	82	83	89
5866-2			122	122	56	56	82	89	83	83
5866-3	124	124	126	126	58	58	82	82	83	83
5866-4	124	124			56	56	82	82	83	83
5866-5	124	124			56	56	82	82	83	89
5866-6	124	124	122	126	56	58	82	82	83	83
**Consensus 5866**	**124**	**124**	**122**	**126**	**56**	**58**	**82**	**82**	**83**	**89**

Legend

Dropout

False allele

NA: low read depth (< 16 reads/locus)

NA: PCR failure (no microsatellite repeat)

References

Brandariz-Fontes C, Camacho-Sanchez M, Vilà C, et al (2015) Effect of the enzyme and PCR conditions on the quality of high-throughput DNA sequencing results. Sci Rep 5:8056. https://doi.org/10.1038/srep08056

Selkoe KA, Toonen RJ (2006) Microsatellites for ecologists: a practical guide to using and evaluating microsatellite markers. Ecol Lett 9:615–629. https://doi.org/10.1111/j.1461-0248.2006.00889.x

Vartia S, Villanueva-Cañas JL, Finarelli J, et al (2016) A novel method of microsatellite genotyping-by-sequencing using individual combinatorial barcoding. R Soc Open Sci 3:150565. https://doi.org/10.1098/rsos.150565

S2 Appendix - File Scripts

Program to calculate the number of reads needed to obtain a correct genotype using Megasat software in the manuscript "Automated genotyping of microsatellite loci from feces with high throughput sequences" by Isabel Salado, Alberto Fernández-Gil, Carles Vilà & Jennifer A. Leonard.

This program is composed by three different scripts, the first calls to the other two by calling them as subprocesses. These scripts were run on Ubuntu 19.04, but if dependencies are satisfied they should be able to run on any Linux or macOS system: Python ≥ 2.7.16, Perl 5.28.1, Biopython 1.74, bash 5.0.3(1), R ≥ 3.5.2, Megasat 1.0.

1_Subsampling.py: Python script to randomly subsample a given number of reads obtained from Illumina MiSeq sequencing of amplicons. We subsampled: 6, 10, 20, 40, 80, 100, 150, 200 reads ("Nreads"). The number of repetitions ("Replicates") for each treatment is 100.

2_Managing_&_Genotyping.bash: bash script to organize files in directories, genotype fastq files using Megasat and prepare format file for the analysis of results in the next script.

3_Comparing_genotypes.R: R script to calculate the proportion of correct genotypes expected for a given number of reads. This script compares genotypes generated in Megasat (in the second script) against reference genotypes. Finally, this script generates a plot which shows the proportion of correct genotypes vs number of reads.

*Notes to run this program:

Sequences are stored in the directory Data/ in a FASTQ format and with the following format name: S*ample-PCR_R1-locus.fastq.*

For Megasat, we have used the following parameters: *number of mismatches = 2, min read depth = 5, max. processors = 1*. To change these parameters, it is necessary to modify line 50 in 2_Managing_&_Genotyping.bash.

Paths in this program are saved in the variables indicated below. To run this program, it is necessary to specify your path for these variables within the different scripts:

- 1_Subsampling.py (lines 28,29): *folder_path_in,* include here your path to directory /Data with all sequences; *folder_path_out,* include here your path to a directory /Sub_output to save new subsampled sequences that will be generated by the program.

- 2_Managing_&_Genotyping.bash (line 62): *inpath,* include here your path to directory /concatenated with concatenated and subsampled fastq files

- 3_Comparing_genotypes.R (line 17-19): *path_in*; include here your path to directory /Megasat_ouput with results of Megasat; *path_out* include here your path to the directory to save final results (i.e. final csv file and graph) created previously in function dir.create().

1_Subsampling.py

```
#!/usr/bin/env python
#-*-coding:utf-8-*-d

#### SUBSAMPLING.PY ####

# Python script to subsample randomly without replacement the number of
next
# generation sequencing reads obtained after a PCR amplification. Nreads
is the
# number of total reads to subsample (treatments: 6,10,20,40,80,100,20O).
# Replicates are the number of repetitions for each treatment (100).
Output
# files are presented in files with prefix 'sub-NreadsX-RepX-'.
Afterwards,
# this script also calls to other additional scripts to genotype and
analyze
# the resulting subsampled fastq files.


## Importing python modules and packages ##

import os
import random
import subprocess
from random import seed
from Bio import SeqIO
from Bio.Seq import Seq
from Bio.SeqRecord import SeqRecord


## Setting the paths where all input files and output files will be
stored. ##

folder_path_in = 'Your path to directory /Data with all fastq files '
folder_path_out = 'Your path to directory /Sub_output to save new
subsampled fastq files'


## Subsampling fastq files: ##

# Setting number of total reads to subsample:
Nreads = [6,10,20,40,80,100,150,200]
# Creating a folder "Sub_output" to save subsampled fastq files:
os.mkdir(folder_path_out)

for i in Nreads:
    replicates = 100
    seed(1)

    # Creating replicates of random number for each file
(replicates=100):
    for j in range(1,replicates+1):
        # Opening files from the path
        for fastq in os.listdir(folder_path_in):
            if fastq.endswith(".fastq"):
```

```
                ruta = folder_path_in + '/' + fastq
                seq_list = list(SeqIO.parse(ruta, "fastq"))
                totseq = len(seq_list)
                # random.sample () subsamples sequences without
replacement
                choice = random.sample(seq_list, i)

                #Writing the output list to a new subsampled fastq file
                ruta_out = folder_path_out + '/' + 'sub-' + 'Nreads' +
str(i) \
                + '-' + 'Rep' + str(j) + '-' + fastq
                SeqIO.write(choice, ruta_out, "fastq")


## Organizing files and running Megasat (calling script in bash): ##
subprocess.call("./2_Managing_and_Genotyping.sh", shell=True)


## Comparing of Megasat results and exporting final results (calling
script in
# R):##
subprocess.call("Rscript 3_Comparing_genotypes.R", shell=True)
```

2_Managing_&_Genotyping.sh

```
#!/bin/bash

#### MANAGING_and_GENOTYPING_GENOTYPING.sh ####

# Bash script to concatenate fastq files by Sample-PCR and creating
folders by
# treatment (Nreads) where concatenated fastq files are moved and where
Megasat
# will be run afterwards



## Creating three variables: ##

# dir = the name of the directory where subsampled fastq files are saved;
dir="Sub_output"

# FILENAME__PCR = a list which keeps part of filenames until PCR to
rename
# concatenated fastqs; Name: sub-Nreads*-Rep*-Sample-PCR
FILENAME_PCR=($(find "$dir" -type f -name '*fastq' | rev | cut -d'/' -f1
\
| rev | cut -d'.' -f1 | cut -d'_' -f1 | uniq))

# DIRNAME_REP = a list which keeps part of filenames until Replicate to
rename
# directories. Name sub-Nreads*-Rep*
DIRNAME_REP=($(find "$dir" -maxdepth 1 -type f -name '*fastq'| rev \
| cut -d'/' -f1 | rev | cut -d'.' -f1 | cut -d'_' -f1 |cut -d'-' -f1-3 \
```

```
| sort -u))



## Concatenating subsampled fastq files by filename (by PCR): ##

# Creating a new directory to save concatenated fastq files
mkdir "$dir"/concatenated
for i in "${FILENAME_PCR[@]}"; do
  cat /dev/null "$dir"/"$i"* > "$dir"/concatenated/"${i[@]}.fastq"
done



## Creating a directory for each treatment and replicate, and moving
# concatenated fastq files (by PCR) to their corresponding directory: ##

for j in "${DIRNAME_REP[@]}"; do
  mkdir "$dir"/concatenated/"${j[@]}"
  mv $(find "$dir"/concatenated/ -name "${j[@]}-*fastq") \
  "$dir"/concatenated/"${j[@]}"
  # Renaming fastq files preparing format of Genotype.txt (output file)
of
  # Megasat:
  rename 's/sub-Nreads\d+-Rep\d+-//' "$dir"/concatenated/"${j}"/*.fastq
  # Running Megasat for each treatment:
  perl MEGASAT_Genotype.pl primer_input_megasat_Mic_coverage.csv 2 5 1 \
  "$dir"/concatenated/"${j}" "$dir"/concatenated/"${j}"
done



## Creating a new directory to copy Megasat's results on it and renaming
each
# file with the name of the treatment and repetition (according to its
# container folder) ##

# Creating a new directory to save Megasat results
mkdir -p Megasat_output || exit 1
inpath='Your path to directory /concatenated with concatenated and
subsampled fastq files'
for l in "$inpath"/*/*/Genotype.txt; do
  cp "$l" "Megasat_output/$( basename "$( dirname "$l")" )_Genotype.txt"
done
```

3_Comparing_genotypes.R

```
##########################################################################
#                         COMPARING_GENOTYPES.R                          #
##########################################################################

# R script to compare the genotypes generated in Megasat of subsampled
fastq
# files with the reference genotypes in order to calculate the number of
needed
# reads to obtain a high proportion of correct genotypes.

## Loading required packages:
library(arsenal)
library(ggplot2)

##### 1. Comparison of genotypes tables. ####

## Creating a variable with input directory (with resulting files from
Megasat)
## and a variable with output directory (with final csv and graph):
path_input <- 'Your path to directory /Megasat_output with results of
Megasat'
dir.create('Your path to the directory /Final_results to save final
results (csv and graph)')
path_output <- 'Your path to the directory to save final results (csv and
graph)
created with dir.create()'

## Creating a df for file with reference genotypes:
reference <- read.delim("Reference_5867genotypes_coverage.csv",
                        header = TRUE, sep = "\t")

## Creating a variable with Megasat output filenames:
files <- list.files(path = path_input, pattern = ".txt",
                    full.names = FALSE, recursive = FALSE)

## Function to compare Megasat ouput with reference:
setwd(path_input)
cmp.lst <- sapply(files, simplify = FALSE, function(x){
  # Read table:
  output_Meg <- read.delim(x, header = TRUE, sep = "\t", stringsAsFactors
= F)
  # Order samples in table by PCR ascending:
  output_Meg <- output_Meg[order(output_Meg$Sample_idx1_idx2), ]
  # Replace "X" and "Unscored" by O:
  output_Meg[output_Meg=="X" | output_Meg=="Unscored"] <- 0
  # Converting characters variables in numeric:
  output_Meg[, 2:23] <- sapply(output_Meg[, 2:23], as.integer)
  # Comparing with reference:
  cmp <- comparedf(reference, output_Meg)
  # Calculate proportion of correct genotypes:
   prop <- 1-((n.diffs(cmp)/132))
   # Prop of correct genotypes = 1 - ((N alelos diferentes/N total alelos
(132))
   # (6 PCR x 11 usat x 2 alelos)
 })
```

```
## Transform list into a data frame:
cmp.df <- data.frame(id=names(cmp.lst), prop=unlist(cmp.lst), row.names =
NULL)
#str(cmp.df)

## Obtaining part of filename through matching with regular expressions:
# Create 2 empty vectors:
Nreads <- c()
Rep <- c()
# Looping through values of list with the filenames:
for (i in files){
  # Strsplit divide the string according to a separator, [[1]][2] select
the
  # position of the list, regexpr("\\d+") select only digital numbers and
  # as.numeric convert str in num.
  value <- as.numeric(regmatches(strsplit(i, '-')[[1]][2],
                                 regexpr("\\d+", strsplit(i, '-
')[[1]][2])))
  # uptdate the vector with new data
  Nreads <- c(Nreads, value)
  value2 <- as.numeric(regmatches(strsplit(i, '-')[[1]][3],
                                  regexpr("\\d+", strsplit(i, '-
')[[1]][3])))
  # uptdate the vector with new data
  Rep <- c(Rep, value2)
}

## Joining new variables (Nreads and Rep) to df:
cmp.df <- cbind(cmp.df, Nreads)
cmp.df.final <- cbind(cmp.df, Rep)

## Writing final table:
setwd(path_output)
write.table(cmp.df.final, file = "depth_prop_correct_genotypes.csv",
            sep = "\t", col.names = TRUE, row.names = FALSE)


#### 2. Graph representation. ####


## Creating a summary table with variables: Nreads, Mean, Se, Min, Max:
# Calculating the descriptive variables:
N <- aggregate(prop~Nreads, FUN = length, cmp.df.final)
MEAN <- aggregate(prop~Nreads, FUN = mean, cmp.df.final)
#Se function
se <- function(x) {
  se <- sd(x, na.rm = T)/sqrt(length(x))
  return (se)
}
SE <- aggregate(prop~ Nreads, FUN = se, cmp.df.final)
MIN <- aggregate(prop~ Nreads, FUN = min, cmp.df.final)
MAX <- aggregate(prop~ Nreads, FUN = max, cmp.df.final)
# Joining variables into a table:
TOTAL = merge(N, MEAN, by = 'Nreads')
TOTAL = merge(TOTAL, SE, by = 'Nreads')
TOTAL = merge(TOTAL, MIN, by = 'Nreads')
```

```
TOTAL = merge(TOTAL, MAX, by = 'Nreads')
names(TOTAL)[2]<-paste("n")
names(TOTAL)[3]<-paste("mean")
names(TOTAL)[4]<-paste("se")
names(TOTAL)[5]<-paste("min")
names(TOTAL)[6]<-paste("max")
TOTAL
# Including observations of 0 values to start the curve in (0,0) in the
graph.
TOTAL <- rbind(c(0,0,0,0,0,0), TOTAL)
TOTAL

## Creating the graph:

# Open pdf file
setwd(path_output)
pdf("depth_rplot.pdf")

# Create the graph
graph <- ggplot(TOTAL, aes(x=Nreads, y=mean)) +
  # setting limits of y axes:
  ylim(0, 1) +
  # setting intervals of x axes
  scale_x_continuous(limits=c(0,200), breaks = c(0,25,50,75,100,150,200))
+
  # marking stablished number of reads in simulations
  geom_point(size = 3.5, shape = 16) +
  # creating a line to connect points
  geom_line(size = 1) +
  # adding a reference horizontal line for y = 0.95:
  geom_hline(yintercept = 0.9, color = "azure4") +
  # establishing axes titles and main title
  xlab("Number of reads") +
  ylab("Proportion of correct genotypes") +
  # setting size, face and distance for axes text and title:
  theme_bw() +
  theme(axis.text = element_text(color = "grey20", size = 12, face =
"plain")) +
  theme(axis.title.y = element_text(color = "black", size = 13, face =
"bold", margin = margin(t = 0, r = 10, b = 0, l = 0))) +
  theme(axis.title.x = element_text(color = "black", size = 13, face =
"bold", margin = margin(t = 10, r = 0, b = 0, l = 0)))

graph

# Close the pdf file
dev.off()
```
